# Atypical Peutz-Jeghers Syndrome Presenting With a Huge Jejunojejunal Intussusception in a Young Male: A Case Report

**DOI:** 10.7759/cureus.36107

**Published:** 2023-03-13

**Authors:** Anas Hashem, Ahmad Ismayl, Amir A Mahmoud, Amani Khalouf, Marwan R Mohammed

**Affiliations:** 1 Internal Medicine, Rochester Regional Health, Rochester, USA; 2 Internal Medicine, Doncaster Royal Infirmary, Doncaster, GBR; 3 General Surgery Department, Al-Qassimi Hospital, Sharjah, ARE

**Keywords:** case report, intestinal hamartomatous polyps, jejunal neoplasms, peutz-jeghers syndrome, intestinal obstruction, intussusception

## Abstract

Intussusception is considered one of the rare causes of intestinal obstruction in adults compared to pediatric patients. It usually presents with non-specific clinical manifestations ranging from mild recurrent abdominal pain to severe acute abdominal pain. The non-specificity of its symptoms makes it difficult to diagnose preoperatively. As 90% of adult intussusceptions are due to a pathological lead point, this prompts the underlying medical condition to be identified. We herein report a rare case of a 21-year-old male with atypical clinical features of Peutz-Jegher syndrome (PJS), presenting with jejunojejunal intussusception as a result of a hamartomatous intestinal polyp. A preliminary diagnosis of intussusception was made after an abdominal computed tomography (CT) scan and was confirmed intraoperatively. Postoperatively, the patient’s condition improved steadily, and he was discharged with a referral to the gastroenterologist for further assessment.

## Introduction

Intussusception is the process where a proximal portion of the intestine becomes telescoped into the lumen of a distal one. This condition can affect peristalsis and cause obstruction as it becomes more difficult for bowel contents to pass through the lumen [1]. Blood flow to the site of the intussusception may also be reduced significantly, resulting in ischemia, necrosis, and possible perforation. One to five percent of all intestinal obstructions in adults are due to intussusception [2]. Of those, 90% have an evident primary cause, while 10% are usually idiopathic. When a primary cause is recognized, a lesion acting as a lead point for the intussusception is usually identified. These lead points can be benign, such as adhesions, Meckel’s diverticulum, and hamartomatous polyps or malignant, like adenocarcinomas, lymphomas, and carcinomas [1]. In adults, an abdominal computed tomography (CT) scan is the modality of choice for diagnosing intussusceptions, and laparotomy with subsequent bowel resection and anastomosis is considered definitive [3]. Herein, we report a 21-year-old male with acute small bowel obstruction because of an unexpected jejunojejunal intussusception caused by a huge hamartomatous Peutz-Jeghers-like polyp.

## Case presentation

**Figure 1:**
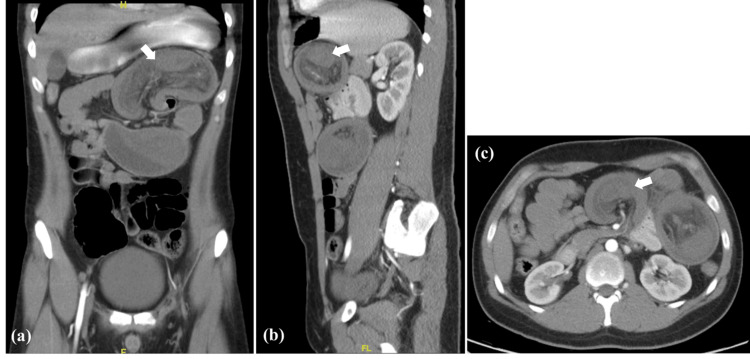
CT scan with oral and IV contrast: (a) coronal view, (b) sagittal view, (c) axial view, all showing dilated proximal jejunal loops (arrows) at the left upper abdomen with configuration of bowel within bowel, indicating the possibility of jejunojejunal intussusception. Congestion of the adjacent mesentery is also present with no passage of oral contrast beyond the intussusception point.

A 21-year-old male presented to the emergency department of our hospital with sharp, colicky epigastric pain of two days duration, radiating to the umbilicus, and associated with one episode of greenish vomitus. History shows similar recurrent episodes of pain aggravated by eating. He denied any episodes of bloody diarrhea or melena, and he was generally a healthy, fit adult with no past medical or surgical history. The patient was tachycardic but hemodynamically stable otherwise. On examination, he was dehydrated, pale, and in severe distress; his abdomen was distended and tense with tenderness in the epigastric and left paraumbilical regions. Exaggerated bowel sounds were present. Blood tests showed an elevated white blood cell count (WBC) with reactive neutrophilia and an elevated C-reactive protein (CRP). Other lab findings, including urea, electrolytes, and lactic acid, were within normal limits. Abdominal ultrasound (US) demonstrated a mass in the epigastric and left upper abdomen. A CT scan of the abdomen with intravenous (IV) and oral contrast was done, showing dilated proximal jejunal loops at the left upper abdomen and a bowel-within-bowel configuration with no oral contrast passage, suggestive of jejunojejunal intussusception and complete intestinal obstruction (Figure 1).

**Figure 2:**
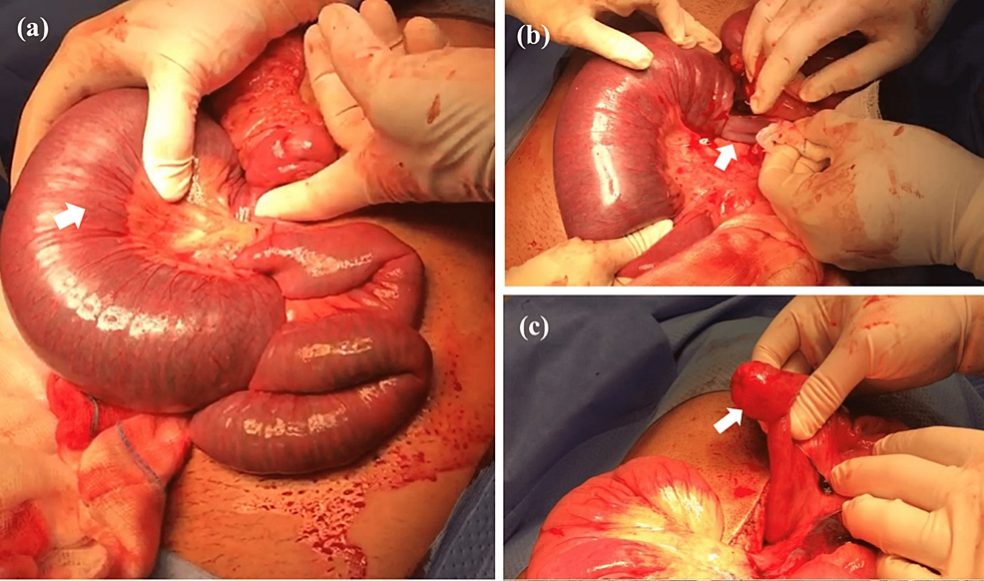
(a) Intraoperative picture showing the jejunojejunal intussusception starting 30 cm from the duodenojejunal junction. Huge dilation of the proximal part of the intestines and engulfing of the mesenteric root inside the intussusception seen (arrow). (b) Careful manual reduction of the intussusception (arrow) and gradual relief of the obstruction. (c) Unveiling of a jejunal intraluminal mass acting as the pathological lead point (arrow) for the intussusception.

**Figure 3:**
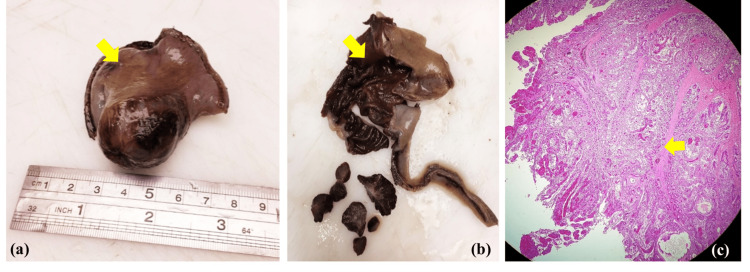
(a) Gross inspection of the resected specimen shows a small intestinal segment (arrow) measuring 6 cm × 4 cm × 4 cm with stapled ends, polypoid intussusception, and gangrenous serosa. (b) Cut surface reveals a 3 cm × 2.5 cm pedunculated polyp (arrow) with hemorrhagic surface and surrounding gangrenous mucosa. (c) The histological view (hematoxylin and eosin, ×100) shows a pedunculated polyp lined by normal glands and crypts of small intestine with goblet cells. There is haphazard arrangement of gland clusters with treelike branching in addition to smooth muscle bundles and fibrosis within the core of the polyp (arrow). The specimen is negative for malignancy.

The patient was resuscitated, and an urgent exploratory laparotomy was performed under the impression of an acute abdomen. A jejunojejunal intussusception 30 cm from the duodenojejunal junction, with dilation of the proximal part engulfing the mesenteric root inside the intussusception. Complete manual reduction of the intussusception revealed a jejunal intraluminal mass (Figure 2). Due to early intervention, only the intussusception segment was congested, while the rest of the bowel was salvaged without ischemia or necrosis. Resection of the intestinal segment containing the intraluminal mass and end-to-end anastomosis were done. Gross inspection of the specimen showed a small intestinal segment with polypoid intussusception and gangrenous serosa. The cut surface of the specimen unveiled a pedunculated polyp within the segment with gangrenous mucosa. Multiple sections under the microscope displayed features indicative of a benign hamartomatous-Peutz-Jeghers-like polyp (Figure 3).

Postoperatively, the patient was hospitalized for eight days due to the consistent passage of loose black tarry stool and low hemoglobin level; therefore, four units of packed red blood cells were transfused. The patient’s condition improved uneventfully and was discharged on the eighth day. Following a thorough medical history and examination, the absence of mucocutaneous pigmentations and a significant family history was insufficient to consider the diagnosis of Peutz-Jeghers syndrome. Therefore, genetic testing was performed and confirmed the diagnosis of atypical PJS, which is de novo in nature. Further follow-up appointments were arranged in the gastroenterology clinic for further assessment.

## Discussion

Intussusception is considered one of the rarest causes of bowel obstruction in adults, showing almost no gender difference, and has an average age at presentation of 50.5 years [4]. With regards to the location, intussusception can be subdivided into enteroenetric (45.5%), ileocolic (34.1%), colocolonic (18.2%), and sigmoidorectal (2.3%) [5]. When considering the etiology, according to Felix et al., 63% of all cases were tumor related. In addition, the malignancy rate was dependent on the location of the mass itself, being more common in the colon (48%) compared to the small bowel (17%) [6]. Our patient developed an enteroenteric intussusception of the jejunum due to a benign hamartomatous polyp.

PJS is considered one of the differential etiologies for the tumor-related causes of intussusception. The syndrome was first described by Peutz in 1921 and Jeghers in 1944 and is characterized by mucocutaneous pigmentation (95%) and hamartomas of the gastrointestinal system [7-9]. It is an inherited autosomal dominant condition with an incidence rate of 1:150,000 individuals. The hyperpigmented macules appear around the mouth and face during infancy [7]. When reviewing 222 patients with PJS, 46.9% had intussusception and 42.8% had obstruction, mostly involving the small intestine [10]. The incidence of developing hamartomatous polyps within the small intestine in PJS is greatest in the jejunum, followed by the ileum and duodenum [11]. The average age of diagnosis was 23 and 26 years in males and females, respectively. According to the World Health Organization (WHO), the diagnostic criteria for PJS include at least one of the following: (1) a minimum of three hamartomatous polyps, (2) a family history of PJS with mucocutaneous pigmentation, (3) a family history of PJS with hamartomatous polyps, or (4) the presence of both hamartomatous polyps and mucocutaneous pigmentation. Therefore, a histological study is necessary to diagnose this syndrome [12]. Hence, our patient’s age of 21 years, the acute onset of jejunojejunal intussusception, and histopathological findings were all suggestive of PJS; however, due to the lack of typical clinical history and physical characteristics of the syndrome, it was almost going to be missed unnoticed. Also, this vague presentation necessitated the performance of genetic testing to confirm a syndrome that is mostly diagnosed clinically.

Since the clinical presentation of patients with intussusception varies and is non-specific, the preoperative diagnosis is challenging (ranging from 32% to 65.8%) [13]. For this reason, it is important to have a well-established approach to confirm the diagnosis in suspected patients. Most patients have complaints of typical small intestine obstruction, so a plain abdominal X-ray is the first modality of choice. This reveals the presence of air-fluid levels with dilated small bowel loops and rules out pneumoperitoneum. Ultrasonography is the gold-standard preoperative diagnostic test for children presenting with intussusception, with an accuracy rate reaching 100%. However, in adults, the efficacy of this test is altered by the presence of distended, gas-filled intestinal loops, lowering the accuracy to around 60% [5]. A "Target" or "Doughnut" sign is seen and is considered pathognomonic for intussusception. The primary diagnostic modality of choice for adults with intussusception is a CT scan of the abdomen, which has a variable diagnostic accuracy reaching up to 100%, a sensitivity of 58-100%, and a specificity of 57-71% [13,14]. A soft tissue mass with a degree of bowel wall edema is seen. Moreover, an eccentric area of fat density around the mass, representing the invaginated mesenteric fat and the mesenteric vessels, is often visible in the CT scan too [15]. In our case, the CT scan with IV contrast displayed intussusception features, and therefore the patient was diagnosed preoperatively.

Regarding management, preoperative hydrostatic reduction is not usually recommended as most of the cases are due to a well-defined pathological lead point. In addition, there is a risk of perforation or the possibility of malignancy [3]. As for the surgical approach, laparotomy is usually preferred as it allows manipulation to adequately visualize the entire small bowel. Initial reduction followed by limited surgical resection is the preferred treatment. Surgical resection without reduction is favored only when underlying primary malignancy is suspected.

## Conclusions

In conclusion, preoperative diagnosis of intussusception is considered challenging due to the non-specific clinical presentation and the lack of an effective diagnostic test. An abdominal CT scan is so far considered the diagnostic modality of choice, and prompt surgical intervention is generally satisfactory in adults. PJS should be considered one of the differential diagnoses in patients with intussusception, with suggestive histological findings of the polyps, even if they do not present with typical PJS features. This will aid in the timely diagnosis of patients with PJS and their management accordingly.
